# Spontaneous coronary artery dissection in a patient with a progestin-releasing intrauterine device

**DOI:** 10.21542/gcsp.2025.23

**Published:** 2025-05-15

**Authors:** Enad Haddad, Sudeep Nugooru, Tyler Lee, Prerana Sevella, Asoka Balaratna

**Affiliations:** 1 Department of Internal Medicine, Jefferson Abington Hospital, Abington, PA, USA; 2 Department of Cardiology, Jefferson Abington Hospital, Abington, PA, USA

## Abstract

Spontaneous coronary artery dissection (SCAD) is a rare cause of acute coronary syndrome (ACS), which predominantly affects women. This case report describes a 55-year-old female with SCAD potentially linked to a progestin-releasing intrauterine device (IUD) placed 1.5 years ago. The patient presented with acute chest pain and diaphoresis, and was eventually found to have ST-elevation myocardial infarction that led to coronary angiography, revealing type 1 SCAD from the proximal right coronary artery (RCA) to the mid-RCA with grade 3 thrombolysis in myocardial infarction (TIMI) flow distally. She was managed conservatively with antithrombotics, statins, and blood pressure control, achieving favorable outcomes without invasive intervention. The IUD was removed due to its potential role as a hormonal trigger. This case underscores the importance of considering SCAD in women presenting with ACS, particularly in those with hormonal risk factors.

## Introduction

Spontaneous coronary artery dissection (SCAD) is a rare cause of acute coronary syndrome (ACS), which is understudied and underdiagnosed. It is not associated with atherosclerosis, iatrogenic injury, or trauma, and its cause is unknown, although it is most likely linked to patient vulnerabilities, inciting triggers, stimulant medications, and hormonal triggers^1^. Given that the vast majority of SCAD patients are women, sex hormones are implicated in SCAD development. However, this relationship remains complicated and unclear, and the underlying mechanisms need to be elucidated^2^. In this report, we discuss the presentation of SCAD associated with the use of an intrauterine device (IUD).

## Case presentation

A 55-year-old African American female with a medical history of peptic ulcer disease and a 20-pack-year smoking history presented to the emergency department for evaluation of an episode of acute chest pain. The patient was walking back from work when she experienced a burning, anterior mid-chest pain that lasted for approximately 10 min and resolved spontaneously. It was associated with diaphoresis and lightheadedness. She experienced a similar episode one month prior to rest, for which she had been taking naproxen 500 mg three times weekly. Additionally, she had a Liletta progestin-releasing (IUD) placed 1.5 years ago. She denied taking any other medications and had no personal or family history of cardiovascular disease.

**Figure 1.**
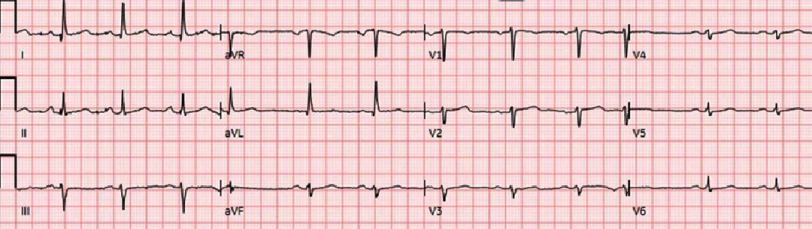
EKG showing normal sinus rhythm with no acute ischemic changes.

In the ED, the patient’s vital signs were within normal limits. Physical examination revealed mild tenderness upon palpation of the anterior chest. Electrocardiography (EKG) showed normal sinus rhythm with no acute ischemic changes (Figure 1). Laboratory results, including troponin and creatinine kinase levels, were within the normal limits. CT angiography of the chest was negative for pulmonary embolism. The patient was admitted for observation via telemetry, and her chest pain was regarded as reproducible, musculoskeletal pain associated with diaphoresis.

**Figure 2.**
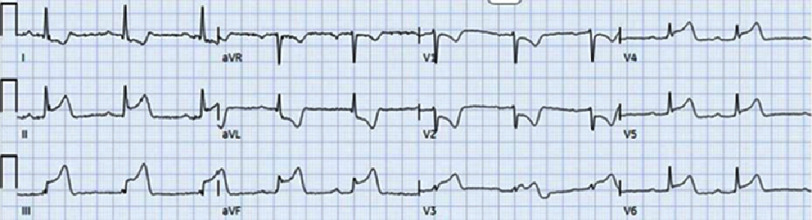
EKG showing ST elevation in leads 2, 3, and avF, with reciprocal depressions in leads V1, V2, and avL.

On the second day after admission, the patient experienced another episode of acute chest pain. EKG showed ST elevation in leads 2, 3, and avF, with reciprocal depressions in leads V1, V2, and avL (Figure 2). The patient received an aspirin load and heparin bolus, and was taken to the catheterization lab.

**Figure 3.**
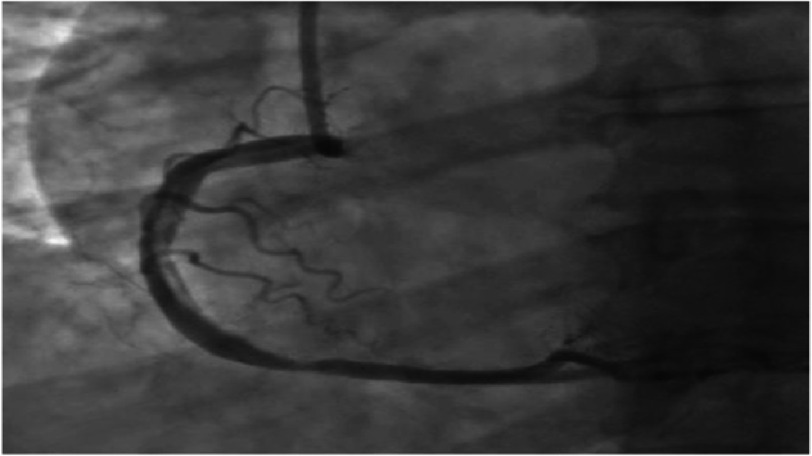
Coronary angiography type 1 SCAD from the proximal RCA to the mid-RCA with TIMI 3 flow distally.

**Figure 4.**
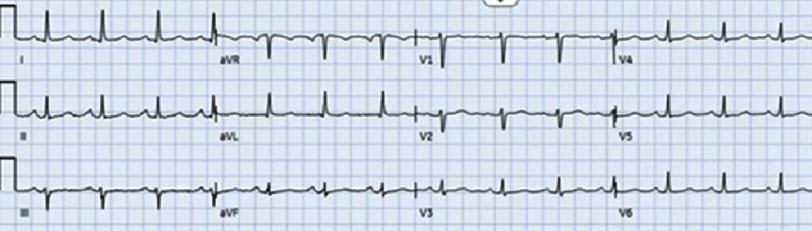
EKG showing complete resolution of previous ischemic changes.

Coronary angiography revealed type 1 SCAD from the proximal right coronary artery (RCA) to the mid-RCA with TIMI 3 flow distally (Figure 3). Given the good flow through and beyond the dissection, percutaneous coronary intervention was aborted, and the patient was treated medically with intravenous heparin, high-dose statins, and aspirin. An IV nitroglycerin drip was used briefly for blood pressure control. EKG performed 1 h after coronary angiography showed complete resolution of the previously observed changes (Figure 4). Coronary artery bypass grafting was considered as an option if the patient developed recurrent ST elevation, which ultimately did not occur.

Transthoracic echocardiography showed an ejection fraction of 60–65% without segmental wall motion abnormalities. Obstetrics and gynecology were involved, and the Liletta progestin-releasing IUD was removed, as it was considered a possible risk factor for SCAD. The patient was eventually discharged in a good condition.

## Discussion

SCAD is a rare cause of acute coronary syndrome (ACS), accounting for 1% of all cases; however, it accounts for 15–20% of myocardial infarction cases during pregnancy and the peripartum period^1^. It is considered an acute coronary event related to a tunica media hematoma that separates the intima from it and compresses the vessel’s lumen^2^. SCAD is similar to atherosclerotic ACS and requires a strong index of suspicion to minimize missed and delayed diagnoses. The vast majority of patients report chest pain with elevated serial biomarkers and ST or non-ST elevation myocardial infarction findings, with stress reported as a precipitating factor^3^. In a retrospective cohort study, it was found that only a fifth of SCAD patients presented with STEMI, with the left anterior descending artery being most involved at 42.4%^4^.

Coronary angiography is the first-line modality for diagnosis given its wide availability and frequent utilization in the management of ACS^5^. SCAD can be classified based on angiographic appearance into type 1 (classic appearance of contrast dye staining of the arterial walls with multiple radiolucent lumens), type 2 (diffuse (usually >20 mm) and smooth stenosis varying from mild stenosis to complete occlusion), and type 3 (hazy or linear stenosis, 11–20 mm in length, and mimics atherosclerosis)^6^. The majority of SCAD cases are treated conservatively, especially in the absence of ongoing ischemia, chest pain, hemodynamic instability, or ventricular arrhythmias. In a Canadian cohort study, 86.4% of 750 patients were treated conservatively with good survival (median hospital stay of 4 days with only one in-hospital death and the majority surviving to discharge)^7^. In terms of outcomes, SCAD was relatively more favorable, with a 1.4% 30-day mortality rate in a retrospective cohort study (vs. 4.1% in non-SCAD ACS cohorts) and a 2.4% one-year mortality compared to 8.8% in the other cohort^4^.

Given SCAD’s predilection for women, hormones are expected to play a role in SCAD; however, direct associations are yet to be established. Possible mechanisms include endovascular alterations or elastic fiber and mucopolysaccharide content. Some studies have suggested that during pregnancy (and peripartum), increased arterial wall shear stress, hemodynamic expansion, and impaired endovascular integrity can contribute to SCAD^8^.

Links between female exogenous hormones and SCAD were documented nearly three decades ago, with Azam et al. reporting a SCAD case associated with oral contraceptive use^9^. The high estrogenic states in combined oral contraceptives have been specifically associated with SCAD, with recommendations leaning towards the use of progestin-only IUD for contraception to decrease the risk of SCAD recurrence^5,10^. However, our patient had a progestin-only IUD placed 1.5 years prior to the onset of SCAD. Nevertheless, HT, including progestin-only IUD, has also been associated with an increased risk of early non-fatal myocardial infarction and unplanned revascularization within 28 days of SCAD onset^11^. This raises the concern that even locally acting progestin-only IUD might play a role in the development of SCAD, possibly secondary to systemic absorption.

Finally, it is important to note that there has been an increase in interest in non-traditional risk factors and their role in cardiovascular diseases in women. These risk factors are divided into three main categories: sex-specific (related to endogenous and exogenous hormones), sex-predisposing (related to diseases that disproportionately affect women, such as autoimmune conditions and migraine), and gender-related (including psychosocial and cultural factors that influence women and their social and physical environment interactions)^12^.

### What have we learned?

SCAD represents an area with a paucity of research exploring and evaluating the effect of non-traditional risk factors discussed above, particularly the use of progestin-only IUDs. Patients at risk of SCAD tend to be younger, female, and lack classical atherosclerotic risk factors, such as hypertension, diabetes, and obesity. SCAD should be considered as a differential diagnosis of chest pain, particularly in women with hormone-related risk factors, including pregnancy, postpartum, HT, and various forms of contraception. Given the increasing evidence of the association between hormonal contraception and SCAD, careful consideration should be given when prescribing these therapies to patients at potential risk, including progestin-only forms of contraception.
